# Syntopic frogs reveal different patterns of interaction with the landscape: A comparative landscape genetic study of *Pelophylax nigromaculatus* and *Fejervarya limnocharis* from central China

**DOI:** 10.1002/ece3.3459

**Published:** 2017-10-04

**Authors:** Vhon Oliver S. Garcia, Catherine Ivy, Jinzhong Fu

**Affiliations:** ^1^ Department of Integrative Biology University of Guelph Guelph ON Canada; ^2^ Present address: Department of Biology McMaster University Hamilton ON Canada

**Keywords:** comparative landscape genetics, isolation by distance, isolation by resistance, life history, microsatellite DNA

## Abstract

Amphibians are often considered excellent environmental indicator species. Natural and man‐made landscape features are known to form effective genetic barriers to amphibian populations; however, amphibians with different characteristics may have different species–landscape interaction patterns. We conducted a comparative landscape genetic analysis of two closely related syntopic frog species from central China, *Pelophylax nigromaculatus* (*PN*) and *Fejervarya limnocharis* (*FL*). These two species differ in several key life history traits; *PN* has a larger body size and larger clutch size, and reaches sexual maturity later than *FL*. Microsatellite DNA data were collected and analyzed using conventional (*F*_ST_, isolation by distance (IBD), AMOVA) and recently developed (Bayesian assignment test, isolation by resistance) landscape genetic methods. As predicted, a higher level of population structure in *FL* (*F*_ST_′ = 0.401) than in *PN* (*F*_ST_′ = 0.354) was detected, in addition to *FL* displaying strong IBD patterns (*r *=* *.861) unlike *PN* (*r *=* *.073). A general north–south break in *FL* populations was detected, consistent with the IBD pattern, while *PN* exhibited clustering of northern‐ and southern‐most populations, suggestive of altered dispersal patterns. Species‐specific resistant landscape features were also identified, with roads and land cover the main cause of resistance to *FL*, and elevation the main influence on *PN*. These different species–landscape interactions can be explained mostly by their life history traits, revealing that closely related and ecologically similar species have different responses to the same landscape features. Comparative landscape genetic studies are important in detecting such differences and refining generalizations about amphibians in monitoring environmental changes.

## 1 INTRODUCTION

Amphibians are often considered excellent ecological indicator species and have been extensively used to monitor environmental quality and habitat fragmentation (e.g., Simon, Puky, Braun, & Tóthmérész, 2011). Numerous studies have demonstrated that both natural and man‐made landscape features form effective genetic barriers to amphibian populations (e.g., Crosby, Licht, & Fu, 2009; Funk, Blouin, et al. 2005; Gibbs, 1998; Lougheed, Gascon, Jones, Bogart, & Boag, 1999). This view of amphibian–landscape interaction is largely drawn from several common amphibian characteristics. Amphibians are highly philopatric, which reduces gene flow and produces large genetic differentiation between subpopulations (e.g., Beebee, 2005; Cushman, 2006; Funk, Blouin, et al. 2005; Murphy, Evans, & Storfer, 2010; Zhan, Li, & Fu, 2009). They also have strict environmental requirements, and movement away from their natal habitats may make them vulnerable to conditions that do not fit in their narrow survival spectrum (Murphy et al., 2010; Stebbins & Cohen, 1995). As these characteristics are found among most amphibian species, this view of amphibian–landscape interaction often forms the foundation for understanding and predicting the effects of landscape on amphibians. While such generalizations are important in understanding how amphibians as a group interact with the landscape, overlooking some important differences between species may produce erroneous predictions. For example, Zhan et al. (2009) failed to detect a significant barrier effect of the Tsinling Mountains, a major divider in the continental East Asia landscape, to Chinese wood frogs (*Rana chensinensis*). Mountain ranges are often perceived as major genetic barriers to amphibian species, and Zhan et al. (2009) suggested that the generalization is likely applicable only to pond breeders and that the Chinese wood frogs are capable of breeding at high‐elevation mountain streams, which likely promotes landscape connectivity between the populations on different sides of the mountain range. Despite many commonalities, each amphibian species may deal with landscape effects differently (e.g., Cushman, 2006).

A better understanding of how species‐specific properties may contribute to species–landscape interaction is essential to establish generalities and to continue to refine such inferences. Comparative landscape genetic analysis that employs multiple species across the same landscape is a powerful approach in working toward this goal. For example, Richardson (2012) compared two co‐occurring amphibian species, the spotted salamander (*Ambystoma maculatum*) and the wood frog (*Lithobates sylvatica*). Contrasting levels of population structure and different response patterns were detected between the two species, despite experiencing the same landscape features. The observed differences were attributed to key differences in movement ability and life history between the two species. Although the number of studies employing this approach is limited, its importance and necessity are gaining wide recognition (e.g., Amos et al., 2012, 2014; Aparicio, Hampe, Fernández‐Carrillo, & Albaladejo, 2012; Engler, Balkenhol, Filz, Habel, & Rödder, 2014; Goldberg & Waits, 2010; Harrisson et al., 2012; Poelchau & Hamrick, 2012). The majority of studies focus on similar impacts of landscape features on distantly related species (e.g., a salamander vs. a frog; Goldberg & Waits, 2010; Harrisson et al., 2012), with few interspecific comparative studies examining the landscape–species interaction on closely related species (Engler et al., 2014).

*Pelophylax nigromaculatus* (Hallowell, 1860) and *Fejervarya limnocharis* (Gravenhorst, 1829) are two ranid frogs (the family Ranidae; Fei et al., 2009) commonly found in continental eastern Asia. In central China, the two species frequently co‐occur in the same habitat (syntopic). Both species are generalists and occupy a wide range of habitats, including small ponds, small‐to‐medium streams, and agriculture land (rice fields). However, they differ by several life history traits, namely body size, clutch size, and time to sexual maturity (Fei et al., 2009). *P*. *nigromaculatus* has a greater average snout–vent length (SVL; males = 62.3 mm, females = 74.4 mm) and a larger clutch size (~3,000 eggs) than *F*. *limnocharis* (SVL males = 40.2 mm, females = 46.0 mm; 700–1,600 eggs). *F*. *limnocharis* reaches sexual maturity in 1 year, while *P*. *nigromaculatus* takes 3 years to reach sexual maturity. These life history traits have important impacts on a species’ demography and dispersal potentials, and they are related to the factors that affect gene flow and may present unique manners of interaction with the landscape. An understanding of these differences between syntopic, closely related species allows us to make *a priori* expectations. Compared to *F*. *limnocharis*,* P*. *nigromaculatus* has a larger body size and therefore likely higher agility, allowing us to predict that this species has low population differentiation and that the landscape would generate low surface resistance. Furthermore, the longer generation time and larger clutch sizes of *P*. *nigromaculatus* suggest a large effective population size (*N*_E_), which would slow changes driven by genetic drift, and hence reduce population substructure.

**Figure 1:**
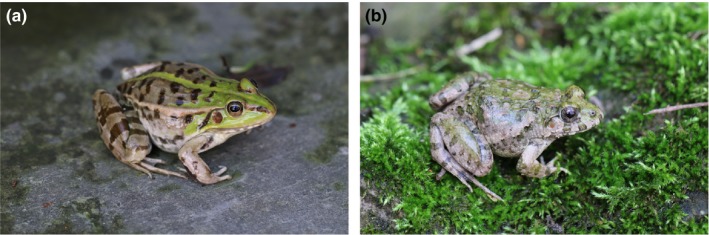
(a) *Pelophylax nigromaculatus*. (b) *Fejervarya limnocharis*. Photographed by Yayong Wu, with permission

In this study, we examine the population genetic structure of *P*. *nigromaculatus* (Figure 1a) and *F*. *limnocharis* (Figure 1b) and landscape features that may have caused the structure. The syntopic condition of the two species guarantees that they have experienced identical landscape configuration. Their close phylogenetic relatedness also reduces potential confounding factors from historical perspective. Minimizing these variables allows us to have a better dissection of landscape–species interaction. We gathered microsatellite DNA data to measure population genetic structure and geospatial data to characterize landscape. The data were then subjected to both classic (e.g., *F*_ST_, AMOVA) and recently developed (assignment tests, isolation‐by‐resistance modeling) landscape genetic analyses.

## 2 MATERIALS AND METHODS

### 2.1 Study area and sampling sites

**Figure 2:**
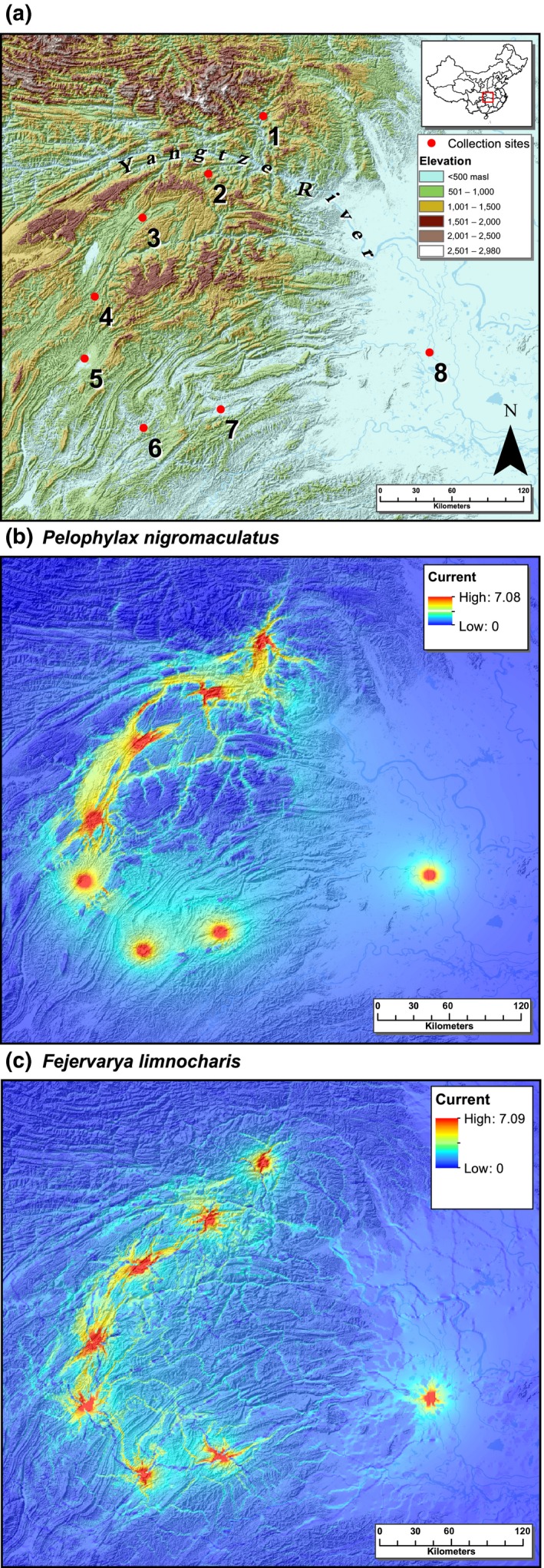
(a) Map of central China showing collection sites for *Pelophylax nigromaculatus* and *Fejervarya limnocharis*. The elevational gradient and the Yangtze River are highlighted which are hypothesized to be relevant landscape features to genetic differentiation. (b) Current map of *Pelophylax nigromaculatus* from isolation‐by‐resistance analysis. (c) Current map of *Fejervarya limnocharis*

Our study area is located in central China, and its landscape structure includes a major river (the Yangtze River) and several of its tributary rivers (e.g., Qing‐Jiang River, Li‐Shui River) as well mountain ranges that separate them (Figure 2a). Agricultural land along the river valleys and roads with medium‐level traffic are also present within the area. Samples from eight sites were collected; while seven sites are located between the mountain ranges and rivers, one (site 8) is further east on the plain, where the landscape is mostly continuous agricultural land (rice field).

Both species are abundant in our study area, and all samples (*P*. *nigromaculatus, n* = 371 and *F*. *limnocharis*,* n* = 432) were obtained from June 6–13, 2008. We aimed at ~50 samples from each site for each species for microsatellite DNA analysis, and for most samples, the two species were collected side by side. One toe from each adult frog was clipped, and the tissue samples were preserved in 95% ethanol and later stored in a −80°C freezer. Detailed sampling site and sample size information is provided in Table 1.

**Table 1:** Collection information for *Pelophylax nigromaculatus* and *Fejervarya limnocharis*

Site	Locality description	Coordinates	Sample size (*n*)	
			*P. nigromaculatus*	*F. limnocharis*
1	Xingshan (XS), Yichang, Hubei Province	N31.33488° E110.76156°	30	57
2	Badong (BD), Enshi, Hubei Province	N30.90124° E110.34852°	57	40
3	Jianshi (JS), Enshi, Hubei Province	N30.61388° E109.72865°	27	72
4	Xuan'En (XE), Enshi, Hubei Province	N29.97850° E109.49275°	63	50
5	Laifeng (LF), Enshi, Hubei Province	N29.51260° E109.41629°	51	52
6	Yongshun YS), Xiangxi, Hunan Province	N28.99019° E109.86053°	50	54
7	Zhangjiajie (ZJJ), Hunan Province	N29.13030° E110.44161°	47	52
8	Lixian (LX), Changde, Hunan Province	N29.558° E112.013°	46	55

### 2.2 Laboratory protocols

Total genomic DNA was isolated using a standard phenol–chloroform protocol (Chomczynski & Sacchi, 1987) and rehydrated in 100 μl of TE buffer (0.01 mol/L Tris‐HCl, 0.001 mol/L EDTA). A total of 16 microsatellite DNA loci (nine for *P*. *nigromaculatus*; seven for *F*. *limnocharis*) were examined using primers developed in this study and previous publications (Aggarwal, Janani, & Sharma, 2012; Gong, Lan, Fang, & Wan, 2010). A summary of the primers used is presented in Appendix S1. Each 25 μl reaction volume contains 1 μl (10–15 ng/μl) of DNA template, 10× TaKaRa Taq™ PCR Buffer (Mg^2+^ free; TaKaRa Biotechnology), 25 mmol/L MgCl_2_, 0.2 mmol/L of each dNTP, 0.75U of TaKaRa Taq™ DNA Polymerase, and 10 μmol/L of each primer with the forward primer labeled with tetrachloro‐6‐carboxy‐fluorescein (TET). Polymerase chain reaction conditions include an initial denaturation step at 95°C for 5 min, then 30 cycles at 95°C for 30 s, primer‐specific annealing temperatures for 30 s (Appendix S1), 72°C for 45 s, and a final extension step of 72°C for 5 min. Amplified allele products were electrophoresed on 6% denaturing polyacrylamide gels and were visualized using an FMBioII^®^ laser scanner (Hitachi). Alleles were scored relative to a TAMRA™ size standard marker (Genescan™ 350, Applied Biosystems) using IMAGE ANALYSIS 3.0 software program (MiraiBio, Inc.).

### 2.3 Summary statistics for genetic diversity

Three indices, number of alleles (*N*_A_), observed heterozygosity (*H*_O_), and expected heterozygosity (*H*_E_), were estimated for each site. Each population was also examined for deviations from Hardy–Weinberg equilibrium using exact test with 1,000,000 Markov chain length and 100,000 dememorization steps. Tests for linkage disequilibrium were conducted between all pairs of loci. All calculations were performed in arlequin 3.1 (Excoffier, Laval, & Schneider, 2005).

We also estimated global and pairwise *F*_ST_. Global *F*_ST_ was used to determine the level of population structure present in each species. Meirmans’ (2006) standardized global *F*_ST_ (*F*_ST_′) was also computed in order to compare between the two species (Hedrick, 2005). The two indices were calculated using genodive 2.0b23 (Meirmans & Van Tienderen, 2004). Pairwise *F*_ST_ (= θ; Weir & Cockerham, 1984) was calculated separately for each species using arlequin with 10,000 permutations.

### 2.4 Genetic clustering

Individual assignment tests were performed for each species to determine the number of naturally occurring genetic clusters within the samples. These tests were conducted using structure 2.3.4 (Pritchard, Stephens, & Donnelly, 2000). We used the admixture model and assumed correlated alleles among populations as these conditions are common to real data. The range of *K* was restricted from 1 to 8, which is the total number of sampling sites. We performed 30 independent runs for each *K* with 500,000 burn‐in periods and 100,000 post‐burn‐in iterations. We used structure harvester (Earl & vonHoldt, 2012) to plot the lnP(D) values against the *K* values and to estimate the delta *K*. The best *K* for each species was determined by considering the trend of ln*p*(D) change over *K*, Delta *K*, as well as individual assignment probabilities.

### 2.5 Analysis of molecular variance (AMOVA)

We conducted locus‐by‐locus AMOVA in order to assess the impacts of landscape features that were hypothesized to contribute to population differentiation. The analysis was performed using arlequin with 10,000 permutations and were evaluated at four hierarchical levels: among groups, among sites within groups, among individuals within sites, and within individuals.

Sampling sites were grouped based on two *a priori* hypotheses. (1) Mountain ranges form significant barriers and populations from the same side of a mountain range would have similar genetic makeup. (2) The Yangtze River is a major barrier to population connectivity. To test the first hypothesis, sampling sites separated by the presence of a mountain range were grouped together, and the eight sites were separated into five groups: (1,2), (3), (4), (5), and (6,7,8). The grouping was determined by the location of each site on a digital elevation map produced by the Consultative Group for International Agricultural Research‐Shuttle Radar Topography Mission (CGIAR‐SRTM). A second analysis with the same grouping but excluding sites 1 and 8 was also conducted. This provides a more stringent evaluation by eliminating the possible compounding effect of the Yangtze River as well as the potential effect of the plain as a site of mixing for all populations (Figure 2a). To test the second hypothesis, two groups were formed by separating the site north of the river (site 1) from the rest of the populations (site 2–8). Similarly, a separate analysis was conducted without site 8.

### 2.6 Isolation‐by‐distance (IBD) analysis

For each species, an IBD analysis was conducted to determine whether geographic distance contributes to the observed population subdivision. The pairwise *F*_ST_/(1 − *F*_ST_) values (Rousset, 1997) were plotted against pairwise straight‐line geographic distances (in kilometers), and the latter were determined using the measure tool in arcmap 10.3.1 (ESRI) and the Universal Transverse Mercator (UTM) projection (Zone 49N). Mantel test was used to determine correlation between the two matrices and was carried out with 10,000 permutations using arlequin.

### 2.7 Isolation‐by‐resistance (IBR) modeling

We conducted an IBR analysis for each species to determine which landscape features significantly impact their genetic connectivity (McRae, 2006). IBR analyses were run in pairwise mode using circuitscape 4.0 (Shah & McRae, 2008), which iterates across all pairs of nodes to produce a pairwise effective resistance distance matrix (McRae, Shah, & Mohapatra, 2013).

We selected five landscape layers in our analysis based on the biology of the study species, including land cover, elevation, water areas, rivers, and roads. Land cover data were obtained from Global Land Cover 2000 Project (GLC2000). Elevation data were obtained through Consultative Group for International Agricultural Research‐Shuttle Radar Topography Mission (CGIAR‐SRTM). All other landscape features were available through the Digital Chart of the World.

To prepare a geospatial map, all layers were projected using UTM Zone 49N and at a cell size resolution of 800 × 800 m. The elevation raster was reclassified into six classes, and each included a 500‐m elevational interval. Road vector data were converted into a raster and were reclassified into two classes (road and trail). The original land cover raster classes were reclassified into seven classes to summarize related land cover types. Water areas and river vector data were converted into raster data and were then mosaicked together with the reclassified land cover layer into a new raster dataset. This modification was to avoid redundancy among similar land cover types and yet include all the information. The resulting mosaic was then classified into nine land cover classes, which joined the pixels matching the Yangtze River and gave inundated land and other minor streams and tributaries their own respective classes (Table 2).

**Table 2:** Landscape resistance parameterization for *P. nigromaculatus* and *F. limnocharis* postoptimization

Landscape feature	*P. nigromaculatus*	*F. limnocharis*
Land cover (nine classes)		
Tree Cover	3	10
Shrub Cover	3	10
Herb Cover	3	10
Regularly flooded areas	1	1
Cropland	1	1
Water (Parent river, inland water)	1,000	1,000
Artificial areas	10	10
Land subject to inundation	1	1
Tributaries/streams	1	1
Road (two classes)		
Road	200	500
Trail	1	1
Elevation, m.a.s.l. (six classes)		
<501	1	1
501–1,000	1	1
1,001–1,500	5	5
1,501–2,000	25	25
2,001–2,500	150	150
2,501–2,980	1,000	1,000

The relative contribution to landscape resistance of each layer (elevation, roads, water, and land cover) was first evaluated separately. Resistance values of each class of each layer were first assigned based on expert opinion (Spear, Balkenhol, Fortin, McRae, & Scribner, 2010) and then optimized by testing a range of biologically informed resistance values (usually 4–10 alternative values). To isolate the landscape feature layer to be evaluated, a resistance value of 1 was given to all other landscape feature layers that are not being tested. Pairwise resistance distances based on the parameterization were obtained using circuitscape, and correlated with pairwise *F*_ST_/(1 − *F*_ST_) using partial Mantel test while controlling for geographic distance. All partial Mantel tests were performed in SPSS statistics 23. The landscape resistance parameterization with the highest correlation coefficient (*r*) was considered optimal, which were subsequently used as basis for additive landscape model (with multiple landscape layers) parameterization.

We generated additive landscape models by combining multiple landscape features to examine their combined effects toward landscape resistance. For simplicity, landscape features were assumed to have additive effects and interaction between layers was assumed minimal. Similarly, pairwise resistance distance matrices were then subjected to partial Mantel tests with pairwise genetic distance controlling for geographic distance.

To serve as baseline for all IBR analyses, we also generated a null IBR model by assigning a resistance value of 1 to all landscape features. This creates an IBD analog that takes into account only the geographic distances between sampling sites, and is expected to provide consistent results with the classic IBD model (Lee‐Yaw, Davidson, McRae, & Green, 2009). As this landscape‐free model is evaluated in the same extent of the finite space defined by the raster map, it allows direct comparison among other IBR analyses over the classic IBD model that considers a boundless landscape (Lee‐Yaw et al., 2009).

All models were ranked according to the strength of correlation. Landscape features that were included in the highest ranking model were considered causal to the observed level of population structure (Cushman, McKelvey, Hayden, & Schwartz, 2006).

## 3 RESULTS

### 3.1 Summary of genetic diversity

For *P*. *nigromaculatus*, all nine microsatellite DNA loci were polymorphic. The number of alleles (*N*_A_) per locus ranged from 2 to 21, and average expected heterozygosity by site (*H*_E_) ranged from 0.455 to 0.769. The two variables were the highest at site #8. For *F*. *limnocharis*, all microsatellite DNA loci were polymorphic except one. The number of alleles for this species ranged from 1 to 21 alleles per locus and the average *H*_E_ by site ranged from 0.255 to 0.447, much lower than these of *P*. *nigromaculatus*. Similarly, the highest *N*_A_ and *H*_E_ occurred at site #8. For both species, most populations for most loci were in Hardy–Weinberg equilibrium. Similarly, most pairs of loci were in linkage equilibrium, and no pair was in disequilibrium for a large number of population pairs. Detailed indices (*N*_A_, *H*_O_, *H*_E_) are presented in Appendices S2 and S3.

### 3.2 *F*_ST_, isolation by distance, and AMOVA

Global *F*_ST_ and standardized global *F*_ST_ (*F*_ST_′) were greater for *F*. *limnocharis* (*F*_ST_ = 0.264; *F*_ST_′ = 0.401) than *P*. *nigromaculatus* (*F*_ST_ = 0.135; *F*_ST_′ = 0.354). Similarly, *F*. *limnocharis* had higher pairwise *F*_ST_ estimates (range: 0.090–0.558) than *P*. *nigromaculatus* (range: 0.037–0.288). Individual pairwise *F*_ST_ estimates are present in Table 3.

**Table 3:** Pairwise *F*_ST_ estimates for *Pelophylax nigromaculatus* (above diagonal) and *Fejervarya limnocharis* (below diagonal)

Site	1	2	3	4	5	6	7	8
1	*	0.18152	0.27708	0.17567	0.14220	0.14785	0.10691	0.12896
2	0.11270	*	0.22335	0.18259	0.19028	0.15449	0.12774	0.07790
3	0.18700	0.18478	*	0.14573	0.27486	0.28841	0.24435	0.18057
4	0.24756	0.20540	0.24224	*	0.10262	0.15857	0.15168	0.13100
5	0.49897	0.43470	0.43642	0.23922	*	0.09379	0.10054	0.12496
6	0.55783	0.49523	0.45974	0.37086	0.26805	*	0.04006	0.06302
7	0.48532	0.40495	0.39918	0.29608	0.22332	0.09020	*	0.03663
8	0.26455	0.18976	0.23483	0.18975	0.22327	0.20146	0.10670	*

**Figure 3:**
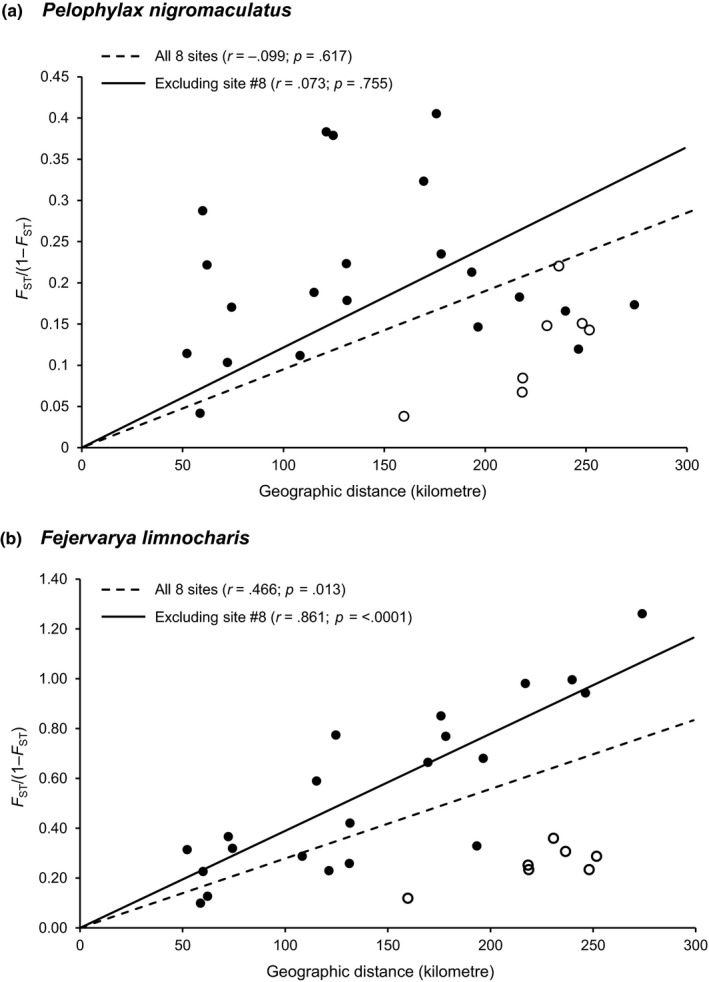
Results from isolation‐by‐distance analysis. Analysis was conducted separately with and without site 8. Open circles represent data points associated with site 8. (a) *Pelophylax nigromaculatus;* (b) *Fejervarya limnocharis*

The IBD analysis revealed a relatively moderate IBD pattern for *F*. *limnocharis* (*r* = .466, *p*_Mantel_ = .013); however, when site 8 was excluded, a strong correlation was observed (*r* = .861, *p*_Mantel_ = <.0001) (Figure 3). In addition, the highest level of population differentiation for *F*. *limnocharis* was observed between sites 1 and 6, which also had the greatest geographic distance between them (274 km). On the other hand, *P*. *nigromaculatus* revealed a very different pattern, and no significant correlation was detected both when all sites were included (*r* = −.099, *p*_Mantel_ = .617) and when site 8 was excluded (*r* = .073, *p*_Mantel_ = .755; Figure 3). The highest level of population differentiation for *P*. *nigromaculatus* was observed between sites 3 and 6, although these sites were not separated by the longest geographic distance. Clearly, geographic distance was not a significant predictor for observed genetic differentiation in *P*. *nigromaculatus*.

Locus‐by‐locus AMOVA showed that, for *P*. *nigromaculatus*, the variation within individuals accounted for the greatest percentage of variation (≈64%; Table 4). Also, mountains contributed to a small but significant percentage of variation. The Yangtze River, however, was not a significant genetic barrier. For *F*. *limnocharis*, the largest variation was among individuals within populations (≈38%; Table 4). All percentages of variation were significant among groups across all grouping schemes. This suggests that, on top of the observed presence of isolation by distance, the intervening landscape features, for example, Yangtze River and mountain ranges, are also causes of genetic differentiation in *F*. *limnocharis*.

**Table 4:** Locus‐by‐locus AMOVA for *P. nigromaculatus* and *F. limnocharis* under two grouping schemes derived from two hypotheses

Hypothesis	Grouping	Variance components	% variation	
			*P. nigromaculatus*	*F. limnocharis*
Mountains as barrier	Sites (1,2); (3); (4); (5); (6,7,8) No. of groups = 5	Among groups	**6.10347**	**16.97049**
		Among populations within groups	**9.13895**	**13.28774**
		Among individuals within populations	**23.77501**	**37.53449**
		Within individuals	**60.98257**	**32.20728**
Mountains as barrier (excl. sites 1, 8)	(2); (3); (4); (5); (6,7) No. of groups = 5	Among groups	**12.92392**	**20.64153**
		Among populations within groups	**3.44289**	**9.44808**
		Among individuals within populations	**19.35973**	**37.03058**
		Within individuals	**64.27345**	**32.87981**
Yangtze River as barrier	(1); (2,3,4,5, 6,7,8) No. of groups = 2	Among groups	2.29648	**12.69474**
		Among populations within groups	**13.86555**	**22.67219**
		Among individuals within populations	**23.51705**	**34.78503**
		Within individuals	**60.32092**	**29.84805**
Yangtze River as barrier (excl. site 8)	(1); (2,3,4, 5,6,7) No. of groups = 2	Among groups	1.80426	**11.59843**
		Among populations within groups	**15.39263**	**26.04363**
		Among individuals within populations	**20.227**	**34.4008**
		Within individuals	**62.57611**	**27.95713**

Bold = significant.

### 3.3 Genetic clustering

**Figure 4:**
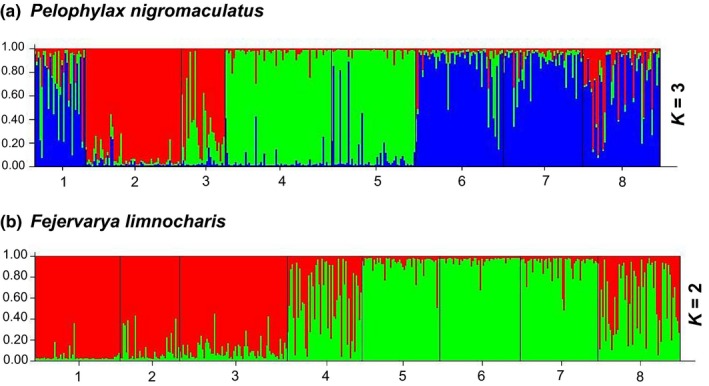
Individual assignment bar plots for (a) *Pelophylax nigromaculatus* and (b) *Fejervarya limnocharis*. Each individual is represented by a vertical bar and its probability of being assigned to a cluster. Numbers at the bottom are site numbers

Individual assignment tests revealed clear genetic clusters within both species. For *P*. *nigromaculatus*,* K* = 3 appeared to be the best‐fit (Figure 4; Appendix S4). Surprisingly, most individuals from sites 1, 6, 7, and 8 formed one genetic cluster. Site 1 locates at the very north, while sites 6, 7, and 8 are at the south (Figure 2a). Most individuals from sites 4 and 5 formed the second cluster, and most individuals from sites 2 and 3 formed the third clusters. In general, there is a large amount of mixing between genetic clusters, particularly in sites 1, 3, and 8.

For *F*. *limnocharis, K* = 2 appeared to be best‐fit (Figure 4; Appendix S5). There was a clear north–south differentiation; while individuals from three northern sites clustered together, individuals from three southern sites were also clustered together. Site 4, located in the middle, was mixed. Site 8, from the eastern plain, was also mixed. Overall, clusters are distinctive, with population in the middle show mixing. This is consistent with the IBD analysis.

### 3.4 Isolation by resistance

Resistance values after landscape feature optimization differed between the two species (Table 2). Optimized resistance values for roads and three land cover classes were higher for *F*. *limnocharis* than for *P*. *nigromaculatus*. Consistent with AMOVA, the Yangtze River was not considered to be a substantial resistant surface for both species. Current maps based on the optimized resistance values are presented in Figure 2b,c.

The landscape resistance models clearly revealed that each species had different causal landscape features that determine their population genetic structure (Table 5). Elevation was the most important causal feature for *P*. *nigromaculatus* (*r* = .702; *p *<* *.0001). In addition, all eight significant models included elevation (Table 5). Roads and land cover were the most important causal feature for *F*. *limnocharis* (*r* = .597; *p *<* *.0001). Also, the top nine best models all included one or both of roads and land cover (Table 5).

**Table 5:** Single landscape feature and additive landscape feature models and the correlations between resistances and genetic distances [*F*_ST_/(1 − *F*_ST_)] for *P. nigromaculatus* and *F. limnocharis*

	*r*	*p*
Landscape model *P. nigromaculatus*		
Elevation	.702	<.0001
Elevation + roads	.687	<.0001
Water + elevation	.672	<.0001
Water + elevation + roads	.637	<.0001
Elevation + land cover	.562	.002
Elevation + roads + land cover	.545	.003
Water + elevation + land cover	.524	.005
Water + elevation + roads + land cover	.497	.008
Land cover	.158	.43
Roads + land cover	.078	.699
Water + land cover	.018	.929
Water + roads + land cover	−.061	.762
IBR–Flat (IBD analog)	−.105	.594
Roads	−.143	.476
Water + roads	−.282	.154
Water	−.329	.093
Landscape model *F. limnocharis*		
Roads + land cover	.597	.001
Elevation + roads + land cover	.592	.001
Water + elevation + roads + land cover	.542	.004
Water + roads + land cover	.498	.008
Land cover	.484	.011
Elevation + land cover	.483	.011
Elevation + roads	.454	.017
Water + elevation + land cover	.424	.027
Water + Land cover	.407	.035
IBR‐Flat (IBD analog)	.394	.038
Water + elevation + roads	.375	.054
Elevation	.256	.198
Roads	.223	.263
Water + elevation	.201	.316
Water + roads	.002	.991
Water	−.652	.000

Correlations between genetic and resistance distances when the landscape was assumed flat were consistent with classic IBD analyses for both species. This was particularly clear for *P*. *nigromaculatus*, which did not have a detectable IBD pattern. Landscape‐induced resistance clearly demonstrated how each landscape feature contributes to the observed genetic differentiation.

## 4 DISCUSSION

### 4.1 Closely related species show divergent patterns of landscape connectivity

The two syntopic ranid frogs clearly displayed different responses to the same landscape, and a summary of contrasting properties between the two species is presented in Table 6. First, *F. limnocharis* exhibited more population differentiation than *P. nigromaculatus*. All indices, including global *F*_ST_, standardized *F*_ST_, as well as pairwise *F*_ST_, support this pattern (Table 3). Although the range of *P. nigromaculatus* extends further north and *F. limnocharis* further south, our study captures the location where the two species overlap and is the center of their distribution. Barring from any particular unknown historical reasons, the observed differences in response patterns between the two species are products of their different interactions with the same landscape. Second, the most noticeable difference, among others, is their response to geographic distance (Figure 3). *F. limnocharis* revealed a clear IBD pattern, and the assignment test also identified two distinctive genetic clusters, one from the south and the other from the north with a mixed population in the middle (Figure 4). On the other hand, *P. nigromaculatus* did not show any of these patterns. Third, the barrier effect of the Yangtze River is complex and interesting. AMOVA detected small but significant effect for *F. limnocharis,* but this is not present for *P. nigromaculatus* (Table 4). Nevertheless, a careful examination of the assignment bar plots (Figure 4) suggests that the barrier effect of the Yangtze River is present for *P. nigromaculatus* and may even be stronger than that in *F. limnocharis*. Most individuals on each side of the river, sites 1 and 2, belong to different genetic clusters in *P. nigromaculatus* (Figure 4). In addition, populations from different sides of the river shared larger pairwise *F*_ST_ in *P. nigromaculatus* than in *F. limnocharis*, despite the fact that the latter species have overall greater pairwise *F*_ST_ values (Table 3). The AMOVA for *P. nigromaculatus* was likely confounded by the shared genetic composition of site 1 with sites 6, 7, and 8 (see further discussion below). Indeed, it is not surprising that the Yangtze River imposes a significant barrier effect on both species as such water bodies are well documented to impede amphibian movement (Lampert, Rand, Mueller, & Ryan, 2003; Lougheed et al., 1999). Finally, IBR analyses also revealed differences in causal landscape features resisting the movement and dispersal of each species, elevation for *P. nigromaculatus* and road and land cover for *F. limnocharis* (Table 5, Figure 2b,c). Throughout all the landscape genetic analysis that we have employed, consistent divergent patterns were shown between the two species despite their close phylogenetic relationship and syntopic condition.

**Table 6:** Summary of contrasting properties between *P. nigromaculatus* and *F. limnocharis*

Property	*P. nigromaculatus*	*F. limnocharis*
Average *H*_E_ by site	0.455–0.769	0.255–0.447
Global *F*_ST_/*F*_ST_′	0.135/0.354	0.264/0.401
Pairwise *F*_ST_ range	0.037–0.288	0.090–0.558
IBD (excl. site 8)	*r* = .073	*r* = .861
AMOVA: Yangtze R. as barrier	No	Yes
Number of genetic clusters (*K*)	3: 1(2) 3 (4,5) (6,7) 8^a^	2: (123) 4 (567)8^a^
IBR, most influential landscape	Elevation	Roads + land cover

a Numbers out of parentheses are mixed populations without clear affiliation.

Most of the differences in the response patterns between the two species can be explained by their distinct life history traits, and the results are consistent with our predictions. With both species collected side by side from the same locations, our sampling design eliminated variables associated with the landscape configuration. These two species differ primarily in several important life history traits that have profound impacts on their migration rate and population size. *P. nigromaculatus* has a much larger body size (male SVL = 62.3 mm, female SVL = 74.4 mm) than *F. limnocharis* (male SVL = 40.2 mm, female SVL = 46.0 mm). A large body size provides high mobility for a species, allows individuals to travel further distances, and is often positively correlated with range size and negatively correlated with regional differentiation (e.g., Hillman, Drewes, Hedrick, & Hancock, 2014; Pabijan, Wollenberg, & Vences, 2012; Wollenberg, Vieites, Glaw, & Vences, 2011). Thus, the reduced population differentiation, diminished IBD pattern, large amount of mixture between genetic clusters, and low landscape resistance values for *P. nigromaculatus* are likely consequences of its large body size. In relation with other life history traits, body size in ranids is positively correlated with clutch size (Gibbons & McCarthy, 1986) and longevity (Morrison, Hero, & Jay, 2004). Indeed, *P. nigromaculatus* is able to produce a larger clutch size (3,000 eggs per clutch) than *F. limnocharis* (700–1,600 eggs per clutch). Large clutch size often promotes juvenile dispersal, which plays a key role in amphibian migration (Funk, Greene, Corn, & Allendorf, 2005). The concentration of a large number of hatchlings in a certain location means intensified resource competition which often selects for offspring dispersal. Thus, juveniles are forced out of their natal sites to relieve such pressure and increase their chances of survival (Hamilton & May, 1977). In addition, large clutch sizes have been documented to lengthen the migration distance in ranid frogs such as in the agile frog (*Rana dalmatina*; Ponsero & Joly, 1998). In combination, a large juvenile population, which is a consequence of a large clutch size driven by a large body size, means a wider coverage of migration and wider geographic ranges as well as a greater fraction of individuals that will survive during the important process of extensive juvenile dispersal (Cooper, Bielby, Thomas, & Purvis, 2008; Cushman, 2006; Funk, Greene, et al. 2005). Furthermore, *P. nigromaculatus* reaches sexual maturity at 3 years, which is much delayed relative to *F. limnocharis* (1 year). Both its large body size and thus longer age to sexual maturity suggest that *P. nigromaculatus* has a longer lifespan and increased fitness (Miaud, Guyétant, & Elmberg, 1999; Miaud, Guyetant, & Helmut, 2000). Due to a longer growth period, *P. nigromaculatus* achieves a larger body size during the time it is capable to reproduce. Consequently, such large body size can produce large offspring that have increased survival rates, in addition to previously mentioned large clutch sizes. Also, having a longer lifespan will likely permit several breeding events allowing the populations of *P. nigromaculatus* to exist in overlaps due to periodic dispersal events following breeding (Marsh & Trenham, 2001; Semlitsch, 2008). The presence of these overlapping generations is known to effectively increase the effective population size (*N*_E_; Nunney, 1993), which slows down genetic drift and lowers population differentiation. This may explain the greater population differentiation in *F. limnocharis* despite our field observations that it appeared to be more abundant than *P. nigromaculatus*. *F. limnocharis* may have a high census population size but low *N*_E_.

Habitat preference and utilization may also contribute to the observed differences. Although both species are considered “generalist” and use a wide range of habitat types, differences in habitat preference do exist. As a fully aquatic species, *P. nigromaculatus* are usually found among valleys, and likely move between habitat patches following available water bodies such as ponds, streams, and river tributaries and distributaries (Fei et al., 2009). Given its large body size and high mobility, the species likely experience low landscape resistance overall, but very high resistance at high‐elevation areas, where water bodies are lacking. This is consistent with our landscape resistance model that high elevations form great resistance for *P. nigromaculatus* and has well‐defined low elevation corridors on the resistance map (Figure 2). Conversely, *F. limnocharis* has flexible habitat associations from semi‐aquatic to primarily terrestrial environments (Fei et al., 2009), allowing greater accommodation in selecting movement corridors with respect to its particular ecological attributes. However, with its small body size and likely limited mobility, *F. limnocharis* experiences high landscape resistance in general, in particular to land cover and roads. Land cover may subject *F. limnocharis* to more biological interactions, such as predation, and environmental stresses, such as desiccation. Desiccation has been noted to be prevalent in small‐bodied amphibians that try to pass through different land cover types due to erratic environmental conditions (Becker, Fonseca, Haddad, Batista, & Prado, 2007; Chelgren, Rosenberg, Heppell, & Gitelman, 2006; Tracy, Christian, & Tracy, 2010).

Site 8 represents an interesting case. It is located on a plain in the east as opposed to the mountainous region of the other sites (Figure 2a). In the study area, the Yangtze River and all its major tributaries run from west to east, and site 8 is located downstream to all other sites. As such, we expect more gene flow from west to east with site 8 receiving genes from all upstream, that is, mountainous sites. This is consistent with our data. For both species, this population (site 8) has the highest genetic diversity (number of alleles, expected heterozygosity; Appendices S2 and S3), and individuals at this site showed mixed genetic affinities (Figure 4). Despite having an average geographic distance farther than any other pairs of sites, its genetic distance is not large (Table 3, Figure 3). Another interesting point is how the eastern plain might have served as a corridor to facilitate population mixing. For *F. limnocharis*, the mixture at site 8 can be explained by mostly downstream dispersal from both southern and northern sites, and site 8 generally receives genes from upstream sites. However, the situation of *P. nigromaculatus* is different. The assignment test placed individuals from site 8 and individuals from the northernmost population (site 1) as well as the two southern‐most populations (sites 6 and 7) into the same genetic cluster (Figure 4). This suggests that the eastern plain may serve as a corridor to facilitate exchange between the northern and southern populations and implies both downstream and upstream dispersal for *P. nigromaculatus*, which is different from patterns observed in *F. limnocharis* (Appendix 5). This possible direction of movement is consistent with our resistance model where the foothill region imposes low landscape resistance to *P. nigromaculatus* (Figure 2b,c).

### 4.2 Species‐specific responses to similar landscape effects emphasize the importance of multiple species comparisons in landscape genetic studies

Our study adds to the limited number of landscape genetic studies that underline interspecific variations among species, an approach suggested to generate more useful information for conservation management (Keller, Holderegger, van Strien, & Bolliger, 2015; Richardson, Brady, Wang, & Spear, 2016). The early cohort of landscape genetic studies have used single species in identifying landscape barriers and generated highly species‐specific management strategies which may not be applicable to other species present in the same environment (Keller et al., 2015; Storfer, Murphy, Spear, Holderegger, & Waits, 2010). Shifting from this approach are current comparative studies that are landscape‐focused (e.g., Amos et al., 2012; Poelchau & Hamrick, 2012), and seek for common impacts of landscape features on distinctly related species occurring in the same landscape. Such studies attempted to identify common elements, such as dispersal corridor, used by multiple species (e.g., Amos et al., 2012), and are particularly useful for land‐use management and identifying priority areas for conservation (Richardson et al., 2016). On the other hand, few studies are species‐focused, which compare closely related species occurring in the same landscape, examine interspecies intrinsic attributes, and seek how species‐specific attributes influence responses to the same landscape (Engler et al., 2014). Species and landscape interaction cannot be limited to the landscape perspective as inherent peculiarities across species influence varying response patterns to the same landscape effects. It is also important to examine inherent variations among species as those that are intrinsically different but share a common landscape may either show similar patterns of interaction with the landscape (e.g., Gagnon & Angers, 2006; Petren, Grant, Grant, & Keller, 2005) or respond uniquely and exhibit distinct patterns despite a shared habitat (e.g., Short & Caterino, 2009; Whiteley, Spruell, & Allendorf, 2004). Thus, a species‐centered comparative approach in conducting landscape genetic studies will refine previous generalizations and will avoid casual association of species with a generic roster of dispersal corridors and landscape barriers. Our study moves forward from simply comparing amphibians, that is, caudates versus anurans (e.g., Richardson, 2012) to demonstrating that interspecific variation, even between phylogenetically and ecologically similar species, may lead to distinct landscape responses and patterns of connectivity. From this template, land‐use management and species conservation applications will be able to integrate species‐specific differences into management decisions and implement refined policies and programs. It will increase the predictive accuracy of generated models as well as efficiency in determining priority among species that need conservation (Murray, Verde Arregoitia, Davidson, Di Marco, & Di Fonzo, 2014).

Amphibians will continue to serve as a chief focal animal group in conducting such landscape genetic studies. Throughout the development of landscape genetics, amphibians are at the forefront for detecting landscape barriers due to their commonly known characteristics of low vagility and high philopatry (Crosby et al., 2009; Cushman, 2006; Murphy et al., 2010; Spear, Peterson, Matocq, & Storfer, 2005). Often these two attributes influence the expectations regarding their patterns of genetic differentiation and prompt assumptions about landscape features that act as barriers to their subpopulations. For example, land‐use management applications usually base their decisions on research that generalize habitat fragmentation and modification as major factors to amphibian decline (Nowakowski, Thompson, Donnelly, & Todd, 2017). However, amphibian species hold many inherent peculiarities that stem from their complex life cycles as well as life history strategies. Evidently, focusing on these species‐specific traits will avoid sweeping generalizations and help refine the established simplifications of amphibian responses to landscape features. Although we cannot perform independent analyses for every amphibian species, identifying those species attributes that likely influence a particular response will help in shaping guided and refined conclusions about species and landscape interactions. If we are able to acquire insight from amphibians with such sensitive and strict survival requirements, it may set a trend in predicting how other taxa respond to landscape effects and provide intuitive information for other more mobile and widespread organisms, for example, birds and mammals.

### 4.3 Future directions

It is important to continue comparative studies that emphasize among‐species variations in order to refine previously established generalizations. The supposed applications and solutions that landscape genetic studies offer to conservation managers often end up in the literature and only a few find actual implementation (Keller et al., 2015). This lag in application of proposed landscape genetic solutions stems from a majority of landscape genetic studies that are conducted for a single species which has limited relevance to conservation management (Segelbacher et al., 2010). Our study has identified and included three life history traits that are intuitively related to a species’ population size and migration rate. These species attributes are discrete and thus can be easily compared and consequently produce predictions about their response to landscape effects. We suggest the inclusion of other intrinsic characteristics that, although possibly with less putative associations to the factors of gene flow, may also play a role in a species’ response to landscape effects. This may involve utilizing species’ ecological attributes which links the species with its habitat in predicting response to landscape effects. Being an important environmental indicator species, one of the major factors that can influence population differentiation in amphibians are the surrounding conditions which often have species‐specific influence (Vernesi et al., 2016). This also indicates the necessity of testing multiple hypotheses for population differentiation as the observed patterns could not be attributed to a single factor. This will enable us to assemble more specifically the suite of inherent species attributes that predict a particular response to landscape effects. From a technical perspective, computer applications that are able to simultaneously optimize multiple environmental parameters, particularly for resistance modeling, will further improve simulation accuracy and the representation of the landscape's heterogeneity.

## 5 CONCLUSIONS

Our comparative analysis of two syntopic amphibian species revealed that closely related and ecologically similar species may have quite different responses to the same landscape features. Although amphibians in general are sensitive to landscape heterogeneity and are good environmental indicator species, significant differences in species–landscape interaction patterns between species should be expected, which has significant implications in predicting organism response to environmental changes or monitoring environmental changes with organism responses. This study also highlights the importance of using multiple species in conducting landscape genetic studies as well as choosing the right species in land‐use management as inherent attributes of organisms influence their response to any particular landscape.

## CONFLICT OF INTEREST

None declared.

## AUTHOR CONTRIBUTIONS

Vhon Garcia collected the genetic data for *Fejervarya limnocharis*, performed data analyses, and drafted the manuscript. Catie Ivy collected the genetic data for *Pelophylax nigromaculatus*. Jinzhong Fu conceptualized and supervised the project and finalized the manuscript. All authors read and approved the manuscript.

## DATA ACCESSIBILITY

All original microsatellite DNA data are provided in Appendices S6 and S7.
