# Supplementary material for: Syntopic frogs reveal different patterns of interaction with the landscape: A comparative landscape genetic study of Pelophylax nigromaculatus and Fejervarya limnocharis from central China
Source: Ecol Evol. 2017 Oct 4;7(22):9294–306. doi: 10.1002/ece3.3459 (PMC5696414; doi:10.1002/ece3.3459)
Supplement: Supplementary file 1 [file ECE3-7-9294-s001.pdf]

Appendix S1. Characteristics of microsatellite loci and primers used for *Pelophylax nigromaculatus* and *Fejervarya limnocharis*

Locus	Primer sequences (5' – 3')	Repeat motif	Ta (°C)	Allele size/range
P. nigromaculatus				
*Rnh-4	F: CGCTTACTATGGGGGGATA	$(GA)_5$	62	154–166
	R: GCCTGAGAAGGGTGGTGCT			
*Rnh-10	F: AGTGCAACATCAACTTGGGTG	$(GCT)_6$	62	165–246
	R: GCAGAGTCGCTGTCGGGA			
*Rnh-9	F: GCACAGTTAGCGAGATGGA	$(GCA)_7$	59	155–185
	R: CTCACTAGAGCTGGGTGGTAT			
*Rnh-6	F: TCTCGGGAGGAAAGCAATGG	$(AAC)_5$	62	208–214
	R: AAGGAGCCTGGGACTATGGTAAAC			
*Rnh-13	F: GATACGGGAGGCAAACG	$(GCA)_5$	56	144–150
	R: TCCACAGCCCAGCACTC			
†JS01-D6	F: TGTGGTCAGATAAGTAGGGT	$(CA)_{11}$	55	152–186
	R: CCAGGACACTTATGTAGGTT			
†JS01-G11	F: GCTCTCCTATGACTCACCC	$(CA)_{15}$	55	132–186
	R: GCAAGCAGTGGTGTAAGTG			
†JS01-B3	F: GTGTGAGTATTTGTATGTGG	$(TG)_{18}$	55	146–178
	R: CAGGAGTATGTAATGAGCAG			
†JS01-A6	F: AGGTTCCTTTAAGGGCTC	$(CA)_{13}$	55	160–182

	R: GGGGTTTCATTTCTGCTG			
F. limnocharis				
^Fej_ccmb_02	F: GGAGGAATGGGGGGGGATGT	$(CA)_{21}$	58	130–156
	R: TGCACACATACACTGACACATACACAAATA			
^Fej_ccmb_03	F: TTCCCTCTCCCTCTTCTCTTCCAC	$(AC)_{9}$	57	194–201
	R: ACGGGATGAATGGGCTGAACTG			
^Fej_ccmb_04	F: TTGCGTTGGTTATGTGTAGGTGC	$(GT)_{30}$	57	87–215
	R: GTCCATTAAAGTACGAAAAACAGTCC			
†LX001-B9	F: AGCATTCAATATCAGGCAGCAG	$(CA)_{17}$	64	164–232
	R: GTGCGCGTGTGTCTTTGTATATA			
†LX001-A7	F: GGGACAGAAATACTGATGATG	$(CA)_{13}$	63	156–170
	R: GTAGACCTTAGAGCAGAAGC			
†LX001-G10-2	F: GCTCATAACAGGCAGCATTG	$(CA)_{13}…(CA)_{9}$	64	188–210
	R: CTTCTCCATTTTGTGACCAG			
†LX001-H10	F: AGAATGTTCCTTGCCCTTTAGG	$(CA)_{14}$	62	132-150
	R: TGACCGTCTGTGTGTGACTGTCAG			

Primers originally developed in this study†, by Gong *et al.* (2010)*, and by Aggarwal *et al.* (2012)^. Ta = annealing temperature.
