# Supplementary material for: Syntopic frogs reveal different patterns of interaction with the landscape: A comparative landscape genetic study of Pelophylax nigromaculatus and Fejervarya limnocharis from central China
Source: Ecol Evol. 2017 Oct 4;7(22):9294–306. doi: 10.1002/ece3.3459 (PMC5696414; doi:10.1002/ece3.3459)
Supplement: Supplementary file 2 [file ECE3-7-9294-s002.pdf]

Appendix S2. Summary statistics of *Pelophylax nigromaculatus* at nine microsatellite loci for eight sampling sites. N_A = number of alleles, H_O = observed heterozygosity, H_E = expected heterozygosity, n = number of samples for each sampling site, P_{HWE} = p value from Hardy-Weinberg equilibrium test

Sampling site	Rhh-4	D6	Rnh-10	G11	Rnh-9	Rnh-6	Rnh-13	B3	A6	Mean (by site)
**Site #1** (n = 30)										
N_A	5	11	5	9	7	2	2	3	6	5.556
H_O	0.633	0.500	0.414	0.500	0.767	0.133	0.000	0.143	0.267	0.373
H_E	0.776	0.810	0.572	0.847	0.667	0.282	0.066	0.543	0.731	0.588
P_{HWE}	0.031	0.000	0.037	0.000	0.560	0.016	0.017	0.000	0.000	
**Site #2** (n = 57)										
N_A	6	10	8	6	5	3	4	6	9	6.333
H_O	0.614	0.482	0.561	0.519	0.554	0.286	0.421	0.351	0.446	0.470
H_E	0.728	0.595	0.568	0.727	0.639	0.528	0.577	0.791	0.851	0.667
P_{HWE}	0.050	0.010	0.188	0.002	0.068	0.000	0.026	0.000	0.000	
**Site #3** (n = 27)										
N_A	4	2	6	7	3	2	2	6	3	3.889
H_O	0.731	0.038	0.360	0.304	0.462	0.250	0.269	0.385	0.240	0.338
H_E	0.673	0.111	0.598	0.499	0.637	0.337	0.238	0.716	0.284	0.455
P_{HWE}	0.038	0.058	0.001	0.001	0.029	0.233	1.000	0.001	0.481	
**Site #4** (n = 63)										
N_A	5	3	10	8	2	4	2	5	7	5.111
H_O	0.698	0.413	0.587	0.542	0.317	0.250	0.435	0.540	0.475	0.473
H_E	0.620	0.405	0.688	0.512	0.311	0.470	0.475	0.768	0.487	0.526
P_{HWE}	0.069	1.000	0.100	0.045	1.000	0.000	0.592	0.000	0.802	
**Site #5** (n = 51)										
N_A	4	5	9	6	3	3	2	9	6	5.222
H_O	0.800	0.360	0.660	0.318	0.510	0.362	0.102	0.400	0.229	0.416
H_E	0.721	0.505	0.810	0.437	0.506	0.488	0.098	0.760	0.412	0.526
P_{HWE}	0.311	0.014	0.042	0.033	1.000	0.084	1.000	0.000	0.001	
**Site #6** (n = 50)										
N_A	5	6	11	9	4	3	2	8	5	5.889
H_O	0.580	0.543	0.596	0.721	0.638	0.341	0.125	0.480	0.447	0.497
H_E	0.677	0.611	0.820	0.802	0.649	0.603	0.189	0.807	0.654	0.646
P_{HWE}	0.036	0.062	0.000	0.016	0.151	0.001	0.064	0.000	0.001	
**Site #7** (n = 47)										
N_A	6	8	15	7	4	3	2	8	6	6.556
H_O	0.652	0.422	0.822	0.659	0.413	0.348	0.341	0.256	0.318	0.470
H_E	0.745	0.608	0.847	0.786	0.620	0.473	0.342	0.840	0.734	0.666
P_{HWE}	0.077	0.018	0.155	0.027	0.000	0.003	1.000	0.000	0.000	
**Site #8** (n = 46)										
N_A	6	11	14	21	5	3	2	10	12	9.333
H_O	0.378	0.262	0.711	0.429	0.614	0.200	0.457	0.261	0.273	0.398
H_E	0.736	0.876	0.860	0.941	0.759	0.541	0.500	0.824	0.881	0.769
P_{HWE}	0.000	0.000	0.009	0.000	0.386	0.000	0.765	0.000	0.000	
**Mean** (by locus)										
N_A	5.125	7	9.75	9.125	4.125	2.875	2.25	6.875	6.75	-
H_E	0.709	0.565	0.720	0.694	0.599	0.465	0.310	0.756	0.629	-

*No pair of loci are in linkage disequilibrium for the majority of populations.
