# Supplementary material for: Syntopic frogs reveal different patterns of interaction with the landscape: A comparative landscape genetic study of Pelophylax nigromaculatus and Fejervarya limnocharis from central China
Source: Ecol Evol. 2017 Oct 4;7(22):9294–306. doi: 10.1002/ece3.3459 (PMC5696414; doi:10.1002/ece3.3459)
Supplement: Supplementary file 3 [file ECE3-7-9294-s003.pdf]

Appendix S3. Summary statistics of *Fejervarya limnocharis* at nine microsatellite loci for eight sampling sites. N_A = number of alleles, H_O = observed heterozygosity, H_E = expected heterozygosity, n = number of samples for each sampling site, P_{HWE} = p value from Hardy-Weinberg equilibrium test

**Collection site**	**Fej_ccmb_03**	**A7**	**Fej_ccmb_04**	**G10-2**	**Fej_ccmb_02**	**H10**	**B9**	**Mean (by site)**
**Site #1** (n = 57)								
N_A	1	1	1	4	2	4	16	4.142857
H_O	N/A	N/A	N/A	0.127	0.018	0.179	0.291	0.087857
H_E	N/A	N/A	N/A	0.475	0.018	0.608	0.882	0.283286
P_{HWE}	N/A	N/A	N/A	0.000	1.000	0.000	0.000	
**Site #2** (n = 40)								
N_A	1	2	1	5	2	3	10	3.428571
H_O	N/A	0.200	N/A	0.111	0.125	0.125	0.611	0.167429
H_E	N/A	0.380	N/A	0.565	0.339	0.522	0.767	0.367514
P_{HWE}	N/A	0.006	N/A	0.000	0.000	0.000	0.013	
**Site #3** (n = 72)								
N_A	1	2	2	9	2	4	8	4
H_O	N/A	0.211	0.082	0.357	0.104	0.211	0.366	0.190257
H_E	N/A	0.359	0.192	0.818	0.223	0.580	0.422	0.370557
P_{HWE}	N/A	0.001	0.001	0.000	0.000	0.000	0.120	
**Site #4** (n = 50)								
N_A	1	2	1	4	2	3	6	2.714286
H_O	N/A	0.100	N/A	0.267	0.042	0.149	0.560	0.159601
H_E	N/A	0.489	N/A	0.655	0.281	0.489	0.659	0.367471
P_{HWE}	N/A	0.000	N/A	0.000	0.000	0.000	0.369	
**Site #5** (n = 52)								
N_A	1	2	1	5	2	5	7	3.285714
H_O	N/A	0.135	N/A	0.100	0.020	0.060	0.314	0.089747
H_E	N/A	0.160	N/A	0.588	0.020	0.627	0.391	0.255084
P_{HWE}	N/A	0.314	N/A	0.000	1.000	0.000	0.034	
**Site #6** (n = 54)								
N_A	1	1	2	4	2	4	10	3.428571
H_O	N/A	N/A	0.019	0.250	0.074	0.037	0.593	0.138947
H_E	N/A	N/A	0.019	0.732	0.138	0.206	0.705	0.257241
P_{HWE}	N/A	N/A	1.000	0.000	0.018	0.000	0.040	
**Site #7** (n = 52)								
N_A	1	1	2	8	2	6	20	5.714286
H_O	N/A	N/A	0.125	0.308	0.019	0.115	0.529	0.156614
H_E	N/A	N/A	0.118	0.614	0.057	0.457	0.919	0.309276
P_{HWE}	N/A	N/A	1.000	0.000	0.029	0.000	0.000	
**Site #8** (n = 55)								
N_A	1	4	3	12	2	7	21	7.142857
H_O	N/A	0.380	0.109	0.479	0.059	0.265	0.373	0.237913
H_E	N/A	0.508	0.105	0.894	0.058	0.629	0.938	0.447317
P_{HWE}	N/A	0.004	1.000	0.000	1.000	0.000	0.000	
**Mean** (by locus)								
N_A	1	1.875	1.625	6.375	2	4.5	12.25	-
H_E	N/A	0.237	0.054	0.668	0.142	0.515	0.710	-

*No pair of loci are in linkage disequilibrium for the majority of populations.

†Hardy-Weinberg equilibrium tests were not performed for monomorphic loci and were marked N/A.
