# Supplementary figures and images for: Syntopic frogs reveal different patterns of interaction with the landscape: A comparative landscape genetic study of Pelophylax nigromaculatus and Fejervarya limnocharis from central China
Source: Ecol Evol. 2017 Oct 4;7(22):9294–306. doi: 10.1002/ece3.3459 (PMC5696414; doi:10.1002/ece3.3459)

**(A)**

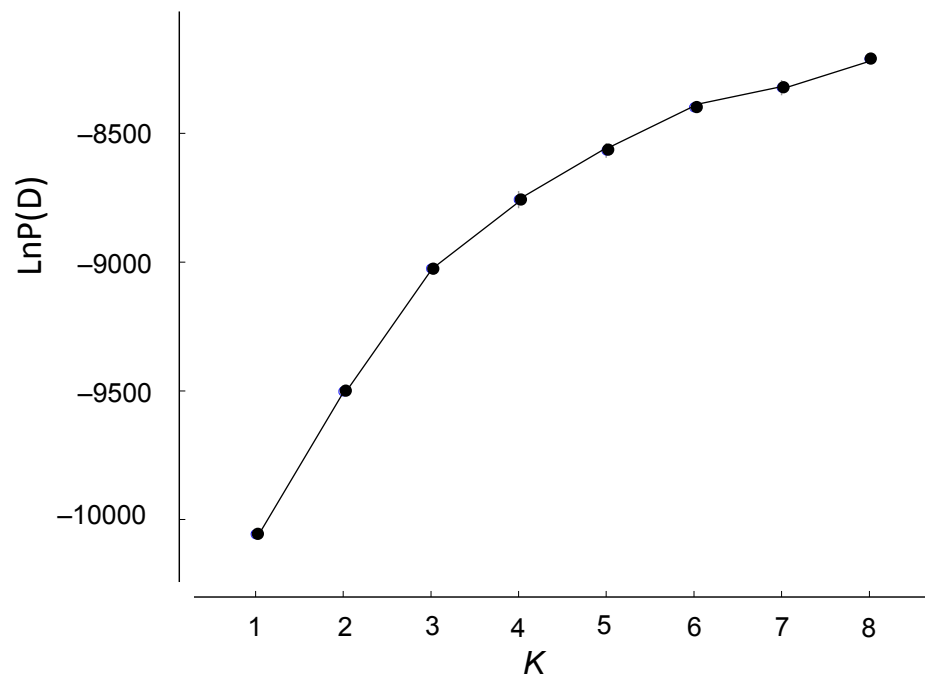

**(B)**

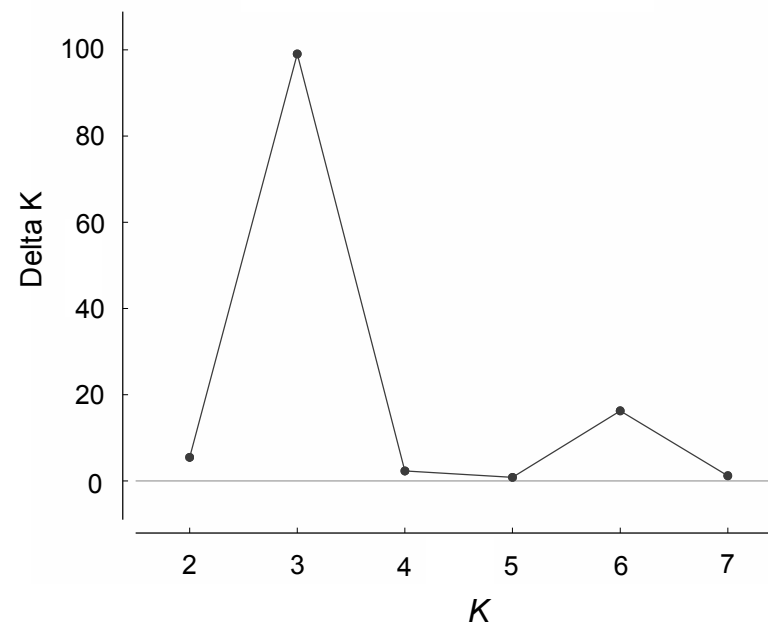

**(C)**

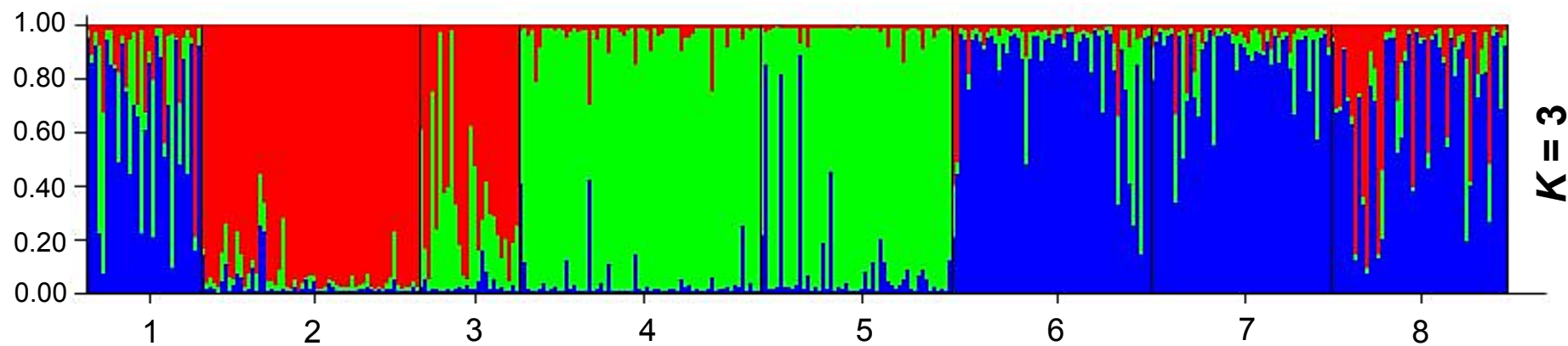

Supplement: Supplementary file 4 [file ECE3-7-9294-s004.pdf]

**(A)**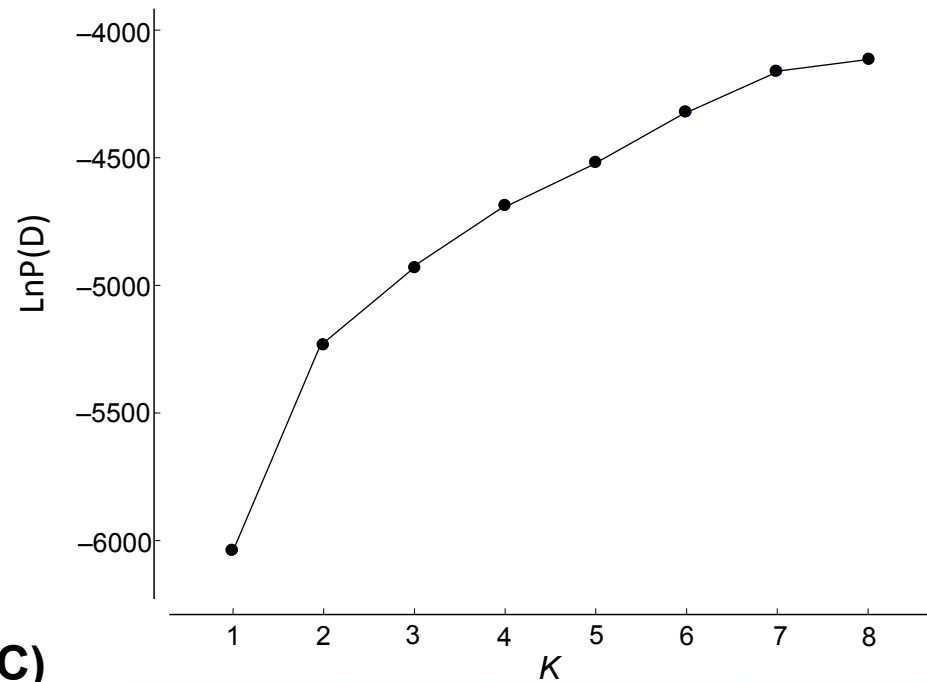**(B)**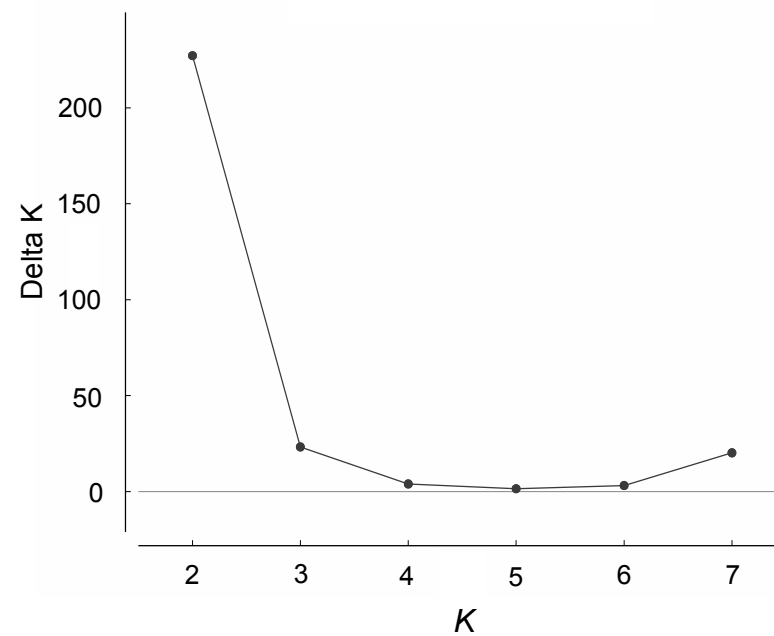**(C)**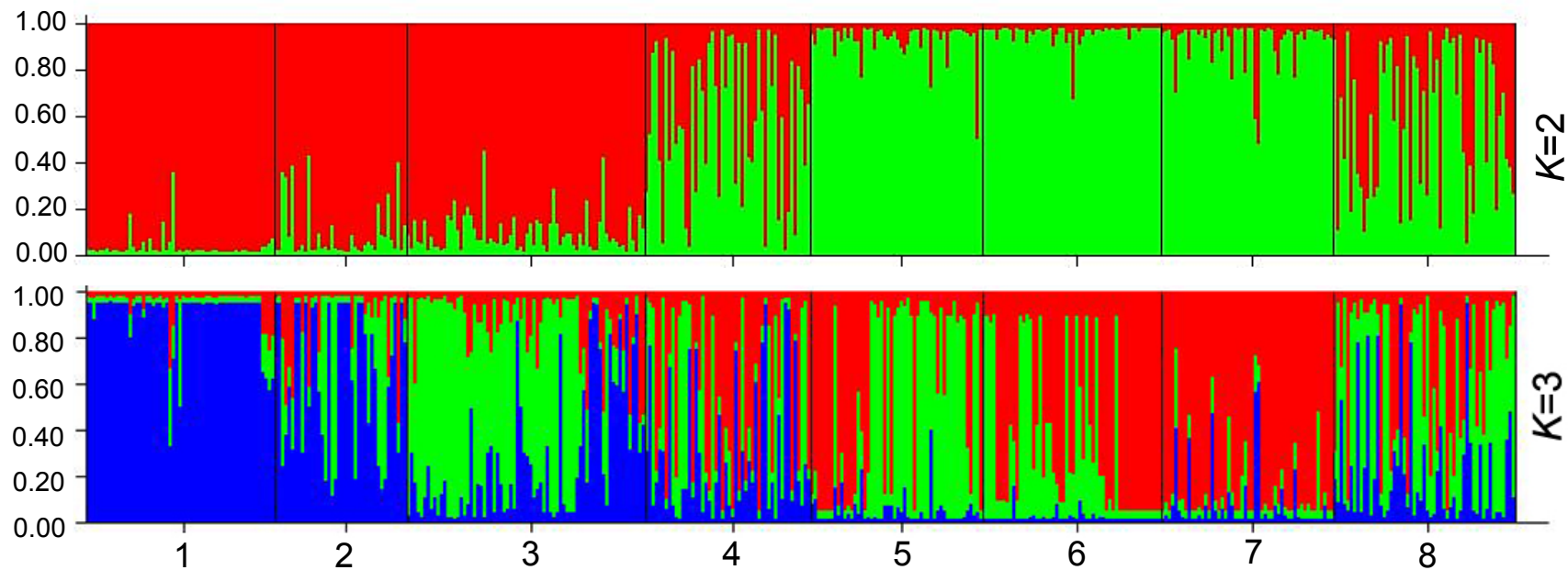

Supplement: Supplementary file 5 [file ECE3-7-9294-s005.pdf]
